# Supplementary material for: Dynamical development of strength and stability of asteroid material under 440 GeV proton beam irradiation
Source: Nat Commun. 2025 Nov 28;16:11710. doi: 10.1038/s41467-025-66912-4 (PMC12753701; doi:10.1038/s41467-025-66912-4)
Supplement: Supplementary file 1 — Supplementary Information [file 41467_2025_66912_MOESM1_ESM.pdf]

Supplementary Information

Dynamical development of strength and stability of asteroid material under 440 GeV proton beam irradiation

Contents

Supplementary Figures

- **Supplementary Fig. 1:** CAD view of the meteorite target envelope and LDV/temperature diagnostic configuration.
- **Supplementary Fig. 2:** List of proton beam shots onto the meteorite sample with corresponding beam parameters.
- **Supplementary Figs. 3–26:** Time-resolved surface displacement profiles of the meteorite sample measured via Laser Doppler Vibrometry (LDV) for beam shots 3–26.
- **Supplementary Figs. 27–50:** Fast Fourier Transform (FFT) spectra of the meteorite sample oscillations corresponding to the individual beam shots.
- **Fig. 51:** Phase boundary of the Campo del Cielo iron meteorite (SEM image and macroscopic photograph).

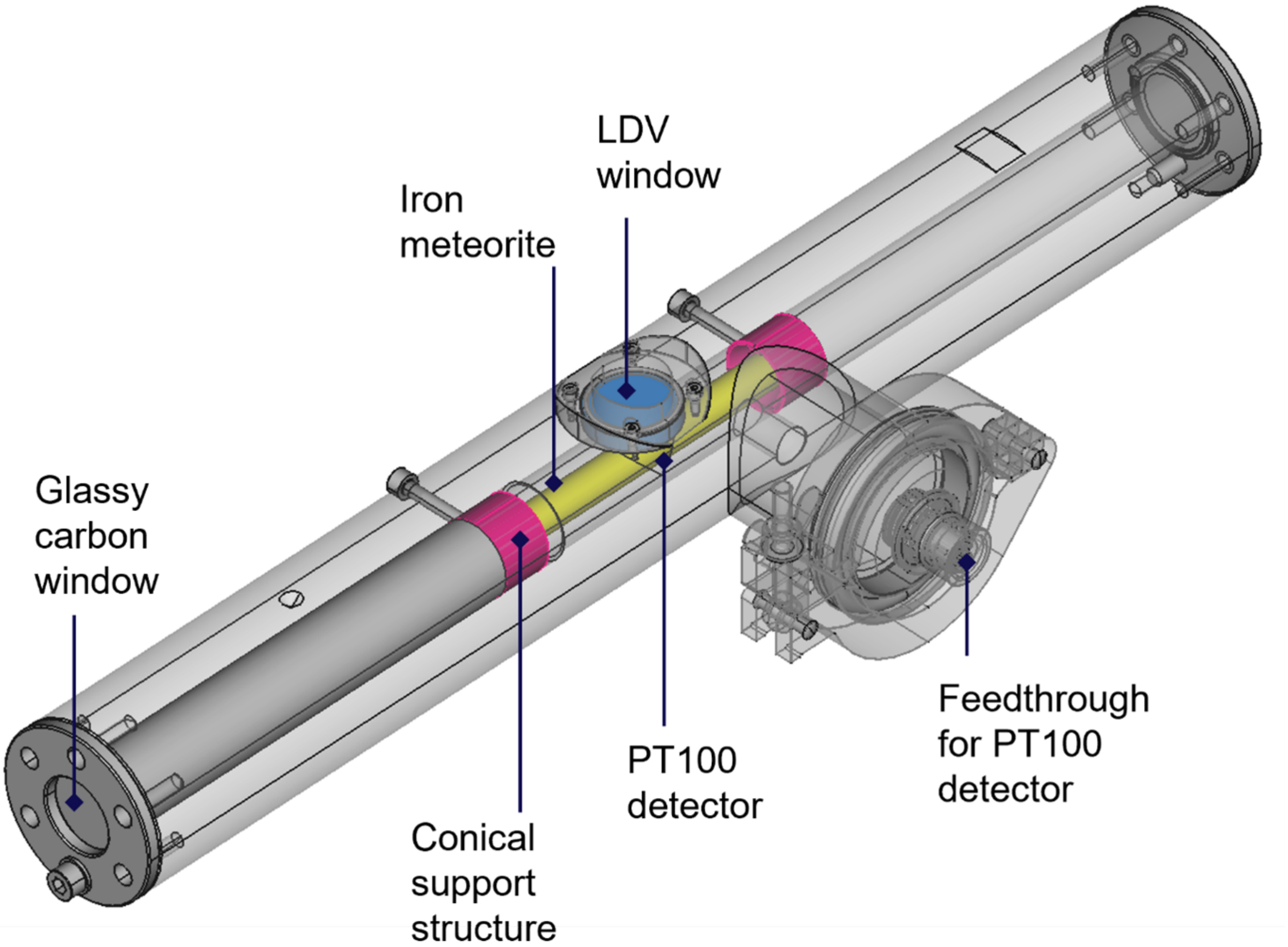

Supplementary Fig. 1: CAD View of Meteorite Envelope. Simplified CAD view of target envelope made of aluminum, with 380 mm length comprising iron meteorite. A conical support structure holding the meteorite sample at its ends allows for free radial oscillations of the sample, measured by a laser Doppler vibrometer (LDV) installed above the sample. The LDV measurement was performed in a perpendicular direction with respect to the p+ beam, utilizing a 1550 nm measurement Laser irradiating through a 5 mm thick UV fused silica high-precision window with an anti-reflection coating (transmissive in the range of 1050 - 1700 nm) that was incorporated in the target envelope. The temperature of the meteorite surface was measured with a 4-wire PT100 platinum resistance thin film detector, attached to the sample on the opposite side of the LDV focal spot.

Beam shot		Beam Intensity		σ_x	Std Dev (σ_x)	R_x^2	σ_y	Std Dev (σ_y)	R_y^2
#	Time	requested	delivered						
1	11:47:09	1×10^{11}	8.52×10^{10}	1.238	0.004	0.996	1.086	0.001	0.998
2	11:48:54	1×10^{11}	9.60×10^{10}	1.225	0.004	0.996	1.076	0.001	0.998
3	11:54:37	1×10^{11}	9.52×10^{10}	1.228	0.004	0.996	1.075	0.001	0.998
4	11:58:35	1×10^{11}	9.65×10^{10}	1.220	0.004	0.996	1.074	0.001	0.998
5	12:26:18	1×10^{11}	9.44×10^{10}	1.237	0.004	0.996	1.085	0.001	0.998
6	12:28:04	1×10^{11}	9.50×10^{10}	1.220	0.004	0.996	1.070	0.001	0.998
7	12:28:57	1×10^{11}	9.47×10^{10}	1.214	0.004	0.996	1.068	0.001	0.998
8	12:29:49	1×10^{11}	9.27×10^{10}	1.211	0.004	0.996	1.065	0.001	0.998
9	12:30:42	1×10^{11}	9.43×10^{10}	1.212	0.004	0.997	1.063	0.001	0.998
10	12:31:35	1×10^{11}	9.39×10^{10}	1.211	0.004	0.996	1.062	0.001	0.998
11/12	14:34:28	3×10^{11}	2.88×10^{11}	1.280	0.002	0.998	1.112	0.001	0.998
13	14:49:25	3×10^{11}	2.86×10^{11}	1.325	0.002	0.998	1.146	0.001	0.998
14	15:02:37	3×10^{11}	2.88×10^{11}	1.324	0.002	0.998	1.146	0.001	0.998
15	15:12:45	3×10^{11}	2.78×10^{11}	1.296	0.002	0.998	1.126	0.001	0.998
16	15:28:09	3×10^{11}	2.88×10^{11}	1.301	0.002	0.998	1.086	0.001	0.998
17	15:47:57	1×10^{11}	1.01×10^{11}	1.262	0.004	0.997	1.092	0.001	0.998
18	15:55:25	1×10^{11}	1.03×10^{11}	1.264	0.004	0.996	1.088	0.001	0.998
19	15:59:49	1×10^{11}	1.02×10^{11}	1.265	0.004	0.997	1.087	0.001	0.998
20	16:12:09	3×10^{11}	2.84×10^{11}	1.328	0.002	0.998	1.152	0.001	0.999
21	16:37:13	3×10^{11}	2.91×10^{11}	1.290	0.002	0.998	1.120	0.001	0.998
22	16:38:06	3×10^{11}	2.81×10^{11}	1.276	0.002	0.998	1.107	0.001	0.998
23	16:38:59	3×10^{11}	2.84×10^{11}	1.273	0.002	0.998	1.104	0.001	0.998
24	16:44:42	3×10^{11}	2.94×10^{11}	1.285	0.002	0.998	1.115	0.001	0.998
25	16:45:35	3×10^{11}	2.86×10^{11}	1.278	0.002	0.999		0.001	0.999
26	16:46:28	3×10^{11}	2.89×10^{11}	1.279	0.002	0.999	1.108	0.001	0.999
27	16:53:57	1×10^{11}	1.02×10^{10}	1.257	0.004	0.997	1.086	0.001	0.998

Supplementary Fig. 2: List of beam shots onto meteorite sample with beam parameters. Size of beam refers to the location of the Beam Television (BTV) and not the location of the experiment. The horizontal size of the beam is in average 10% smaller at the experimental location compared to the BTV.

Supplementary Figures 3–26 present the time-resolved displacement profiles obtained through Laser Doppler Vibrometry for the corresponding beam shots.

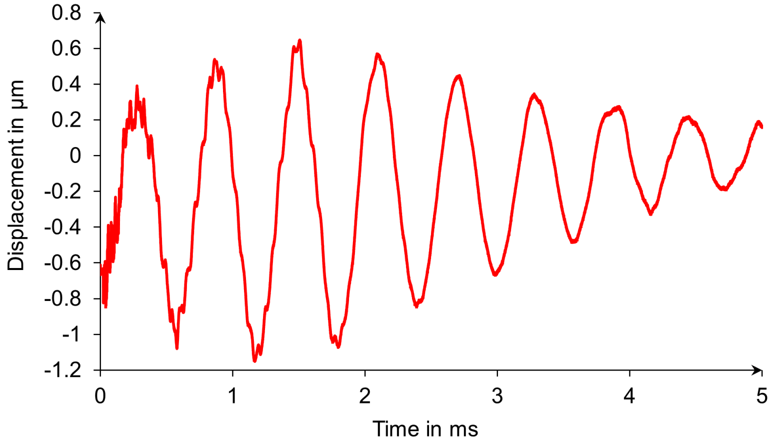

Supplementary Fig. 3: Surface displacement of the meteorite sample measured by LDV. The plot shows the displacement (in µm) as a function of time (in ms) for shot 3 with a beam intensity of 1×10^{11} protons.

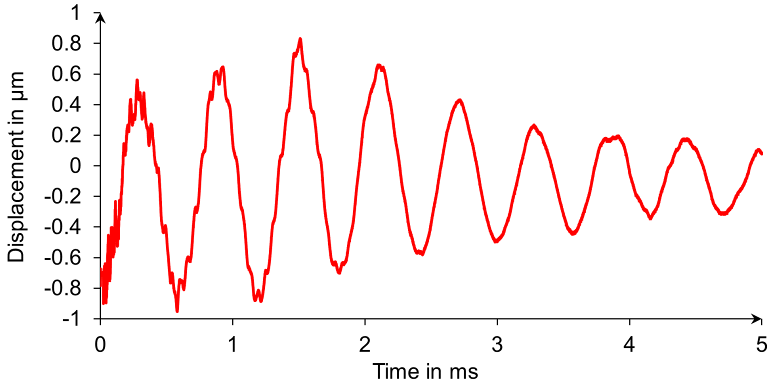

Supplementary Fig. 4: Surface displacement of the meteorite sample measured by LDV. The plot shows the displacement (in µm) as a function of time (in ms) for shot 4 with a beam intensity of 1×10^{11} protons.

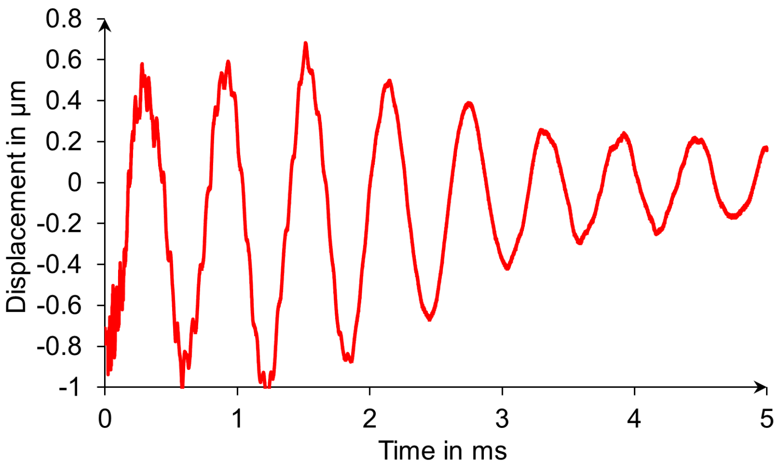

Supplementary Fig. 5: Surface displacement of the meteorite sample measured by LDV. The plot shows the displacement (in µm) as a function of time (in ms) for shot 5 with a beam intensity of 1×10^{11} protons.

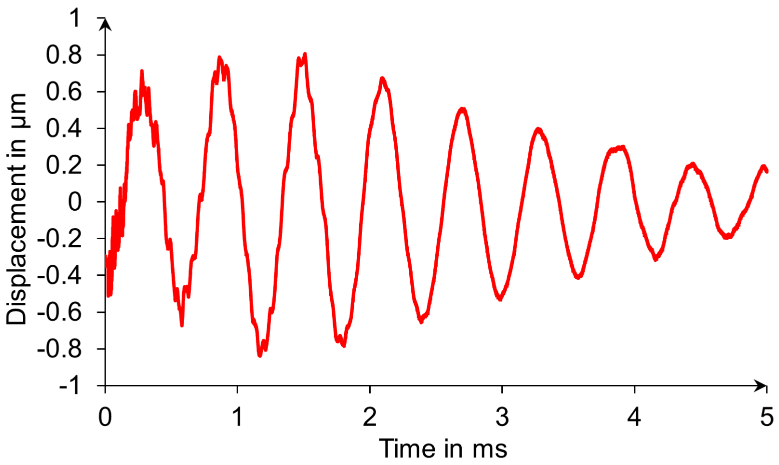

Supplementary Fig. 6: Surface displacement of the meteorite sample measured by LDV. The plot shows the displacement (in µm) as a function of time (in ms) for shot 6 with a beam intensity of 1×10^{11} protons.

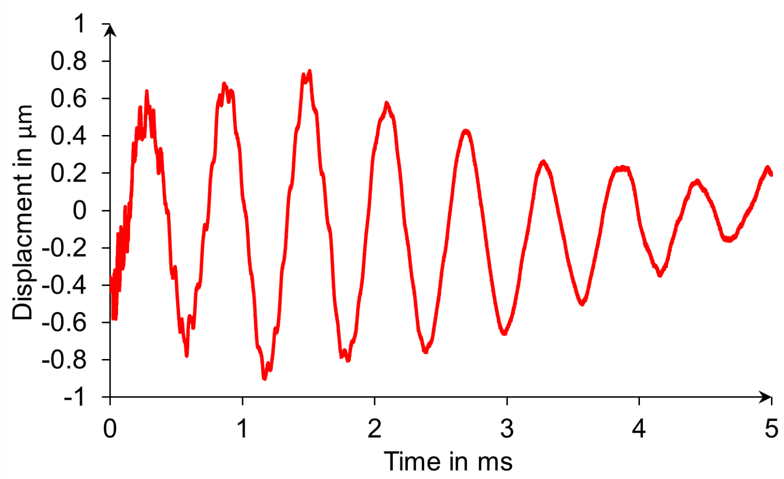

Supplementary Fig. 7: Surface displacement of the meteorite sample measured by LDV. The plot shows the displacement (in µm) as a function of time (in ms) for shot 7 with a beam intensity of 1×10^{11} protons.

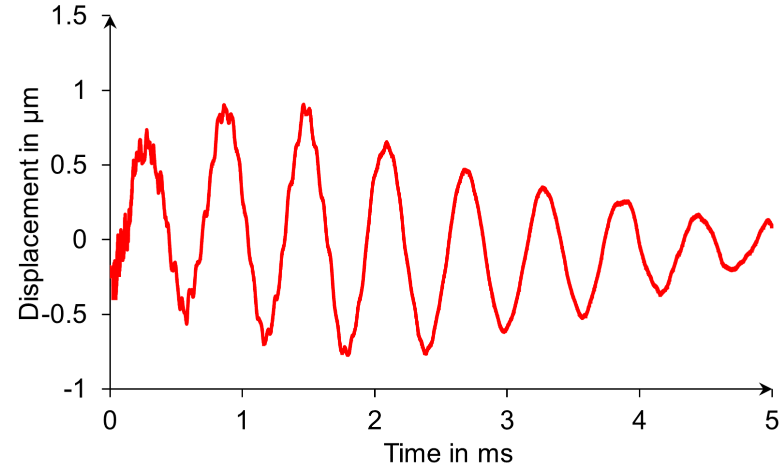

Supplementary Fig. 8: Surface displacement of the meteorite sample measured by LDV. The plot shows the displacement (in µm) as a function of time (in ms) for shot 8 with a beam intensity of 1×10^{11} protons.

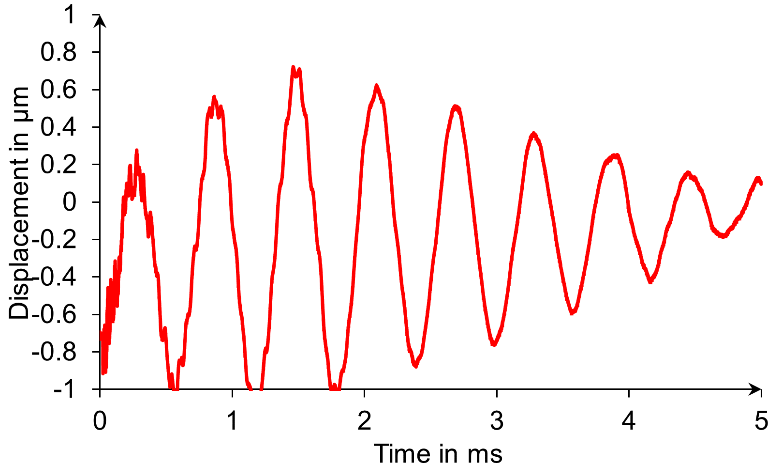

Supplementary Fig. 9: Surface displacement of the meteorite sample measured by LDV. The plot shows the displacement (in µm) as a function of time (in ms) for shot 9 with a beam intensity of 1×10^{11} protons.

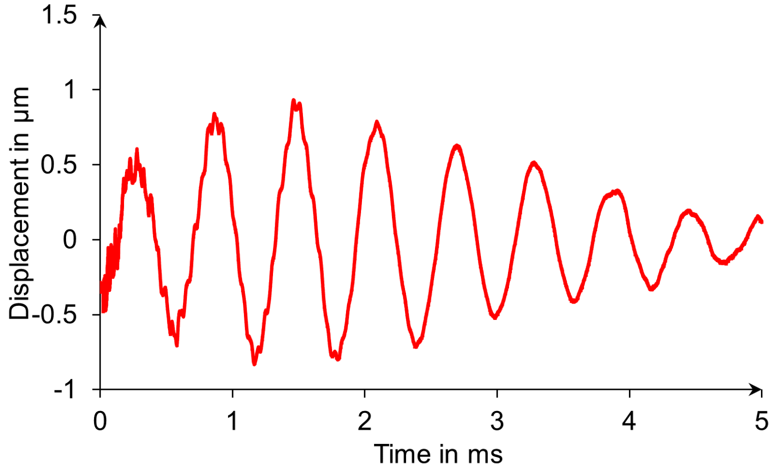

Supplementary Fig. 10: Surface displacement of the meteorite sample measured by LDV. The plot shows the displacement (in µm) as a function of time (in ms) for shot 10 with a beam intensity of 1×10^{11} protons.

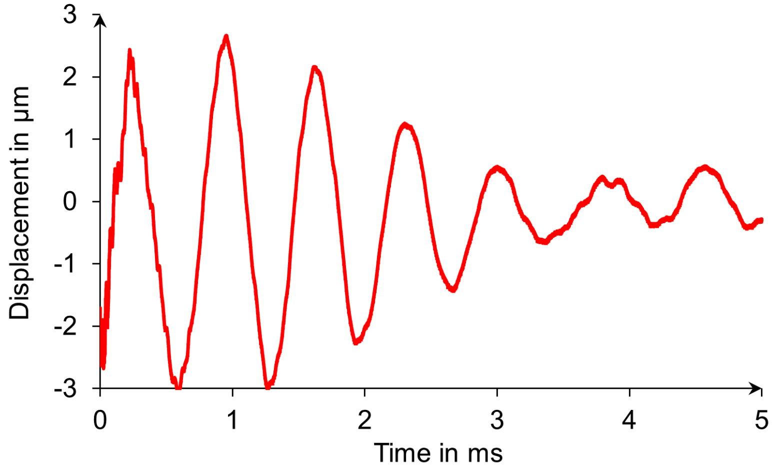

Supplementary Fig. 11: Surface displacement of the meteorite sample measured by LDV. The plot shows the displacement (in µm) as a function of time (in ms) for shot 11&12 (with a beam intensity of 3×10^{11} protons.

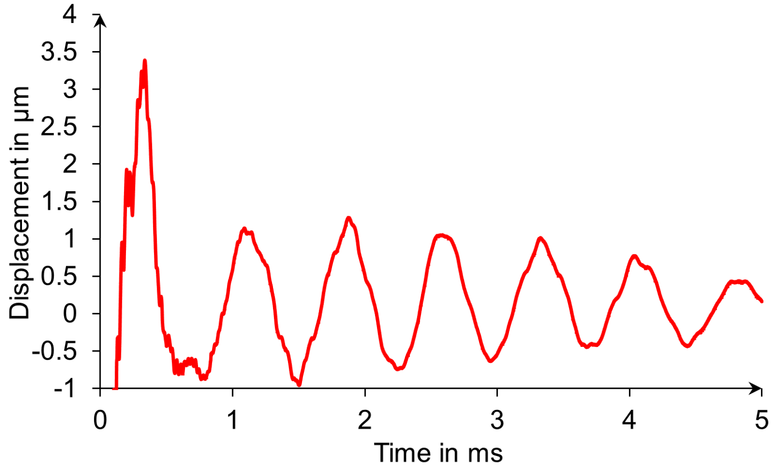

Supplementary Fig. 12: Surface displacement of the meteorite sample measured by LDV. The plot shows the displacement (in µm) as a function of time (in ms) for shot 13 with a beam intensity of 3×10^{11} protons.

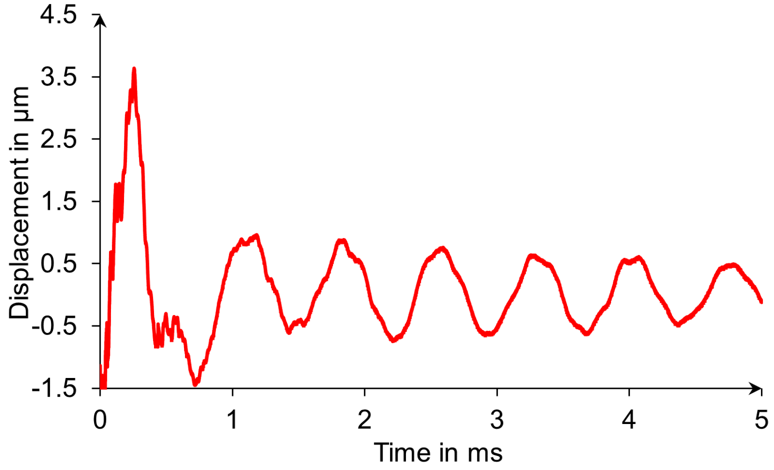

Supplementary Fig. 13: Surface displacement of the meteorite sample measured by LDV. The plot shows the displacement (in µm) as a function of time (in ms) for shot 14 with a beam intensity of 3×10^{11} protons.

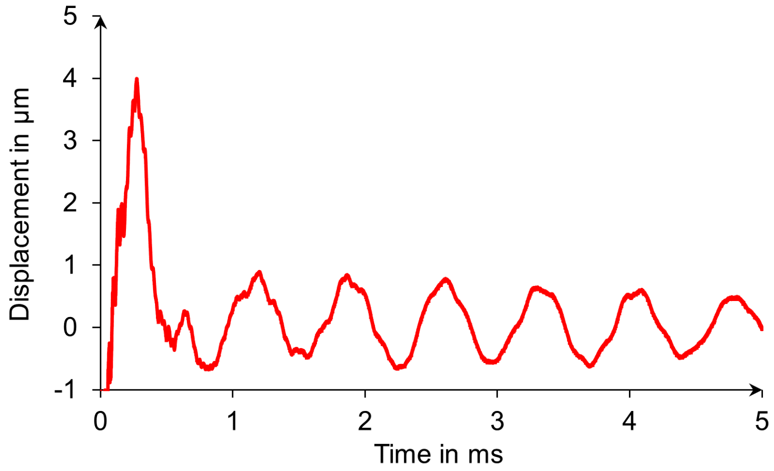

Supplementary Fig. 14: Surface displacement of the meteorite sample measured by LDV. The plot shows the displacement (in µm) as a function of time (in ms) for shot 15 with a beam intensity of 3×10^{11} protons.

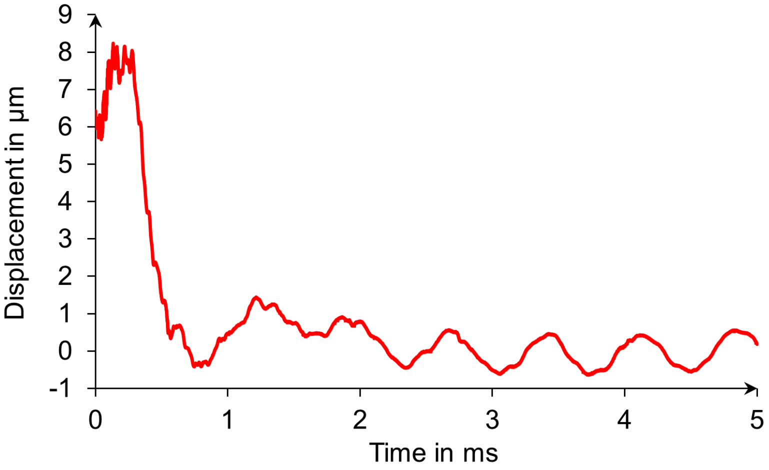

Supplementary Fig. 15: Surface displacement of the meteorite sample measured by LDV. The plot shows the displacement (in µm) as a function of time (in ms) for shot 16 with a beam intensity of 3×10^{11} protons.

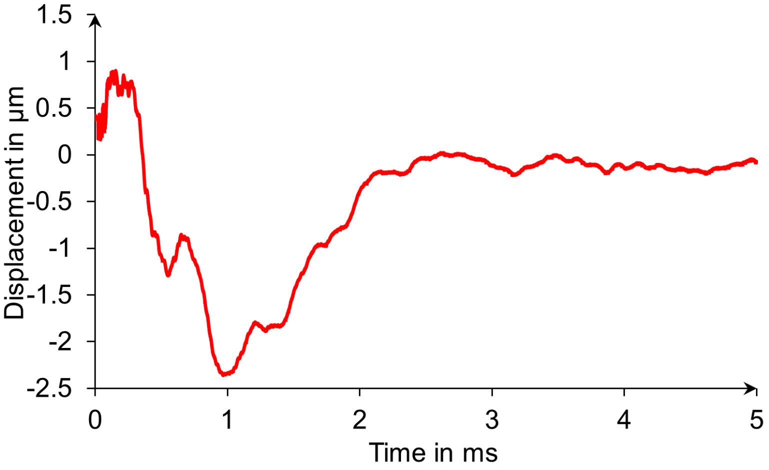

Supplementary Fig. 16: Surface displacement of the meteorite sample measured by LDV. The plot shows the displacement (in µm) as a function of time (in ms) for shot 17 with a beam intensity of 1×10^{11} protons.

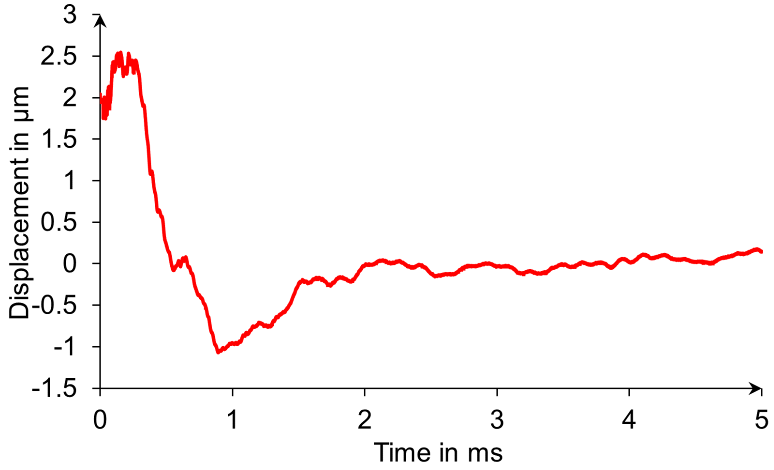

Supplementary Fig. 17: Surface displacement of the meteorite sample measured by LDV. The plot shows the displacement (in µm) as a function of time (in ms) for shot 18 with a beam intensity of 1×10^{11} protons.

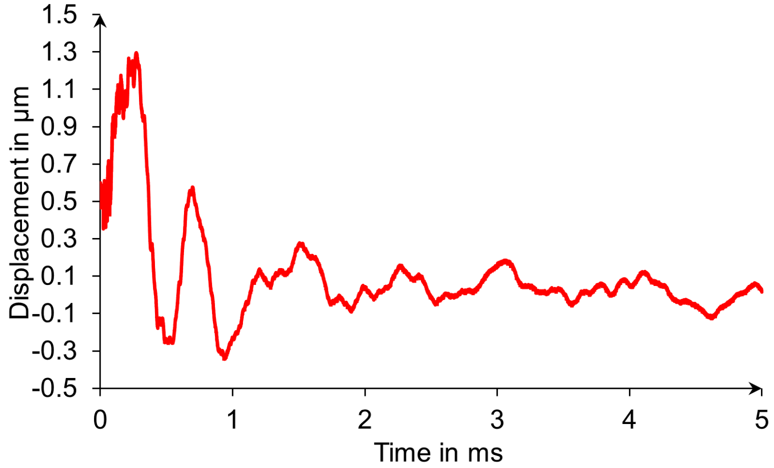

Supplementary Fig. 18: Surface displacement of the meteorite sample measured by LDV. The plot shows the displacement (in µm) as a function of time (in ms) for shot 19 with a beam intensity of 1×10^{11} protons.

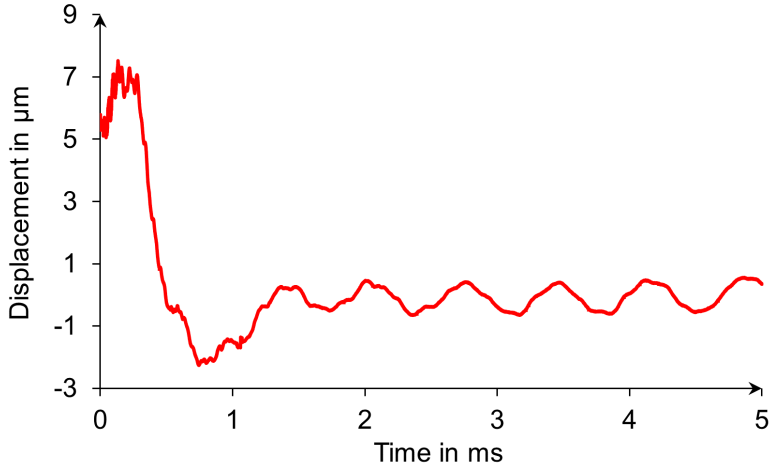

Supplementary Fig. 19: Surface displacement of the meteorite sample measured by LDV. The plot shows the displacement (in µm) as a function of time (in ms) for shot 20 with a beam intensity of 3×10^{11} protons.

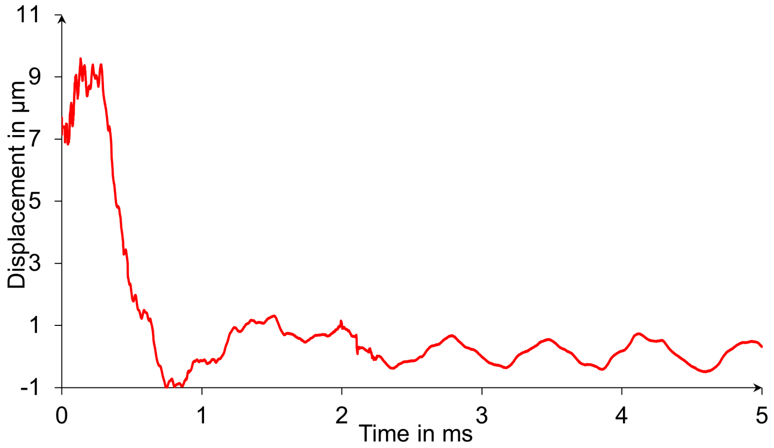

Supplementary Fig. 20: Surface displacement of the meteorite sample measured by LDV. The plot shows the displacement (in µm) as a function of time (in ms) for shot 21 with a beam intensity of 3×10^{11} protons.

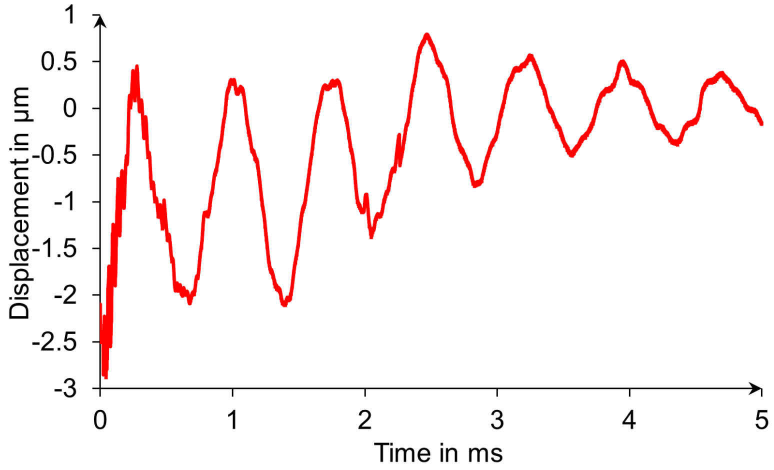

Supplementary Fig. 21: Surface displacement of the meteorite sample measured by LDV. The plot shows the displacement (in µm) as a function of time (in ms) for shot 22 with a beam intensity of 3×10^{11} protons.

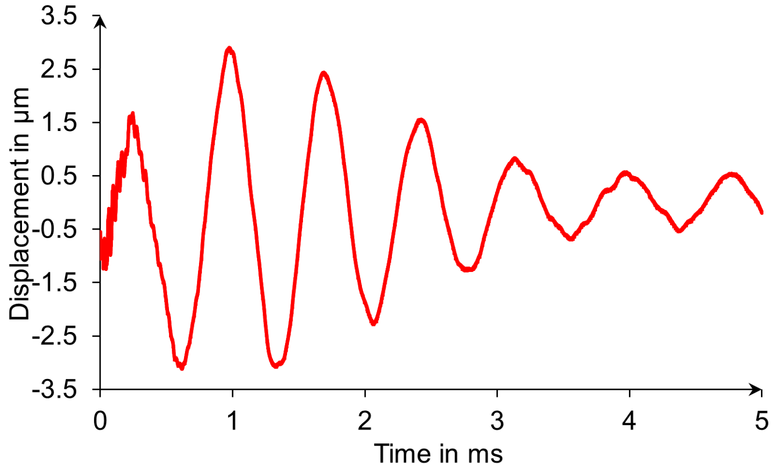

Supplementary Fig. 22: Surface displacement of the meteorite sample measured by LDV. The plot shows the displacement (in µm) as a function of time (in ms) for shot 23 with a beam intensity of 3×10^{11} protons.

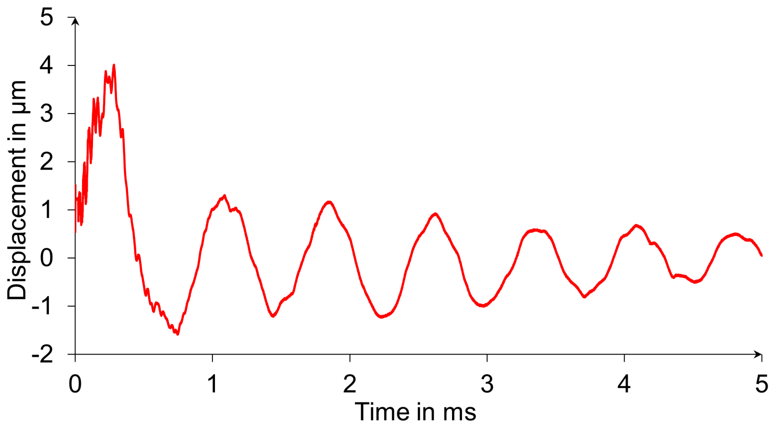

Supplementary Fig. 23: Surface displacement of the meteorite sample measured by LDV. The plot shows the displacement (in µm) as a function of time (in ms) for shot 24 with a beam intensity of 3×10^{11} protons.

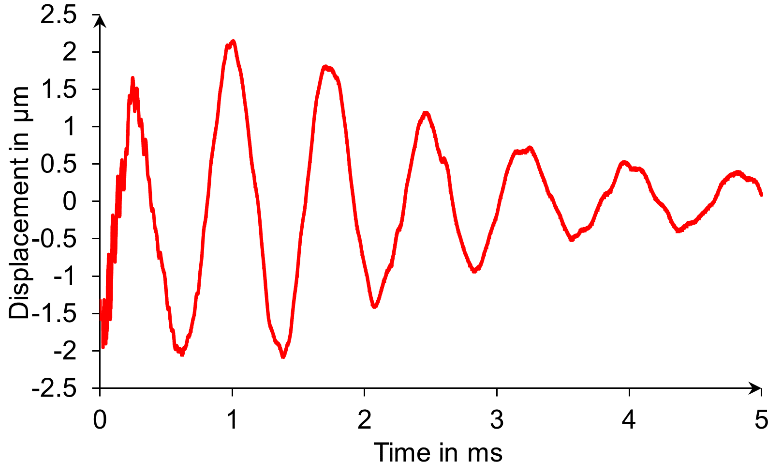

Supplementary Fig. 24: Surface displacement of the meteorite sample measured by LDV. The plot shows the displacement (in µm) as a function of time (in ms) for shot 25 with a beam intensity of 3×10^{11} protons.

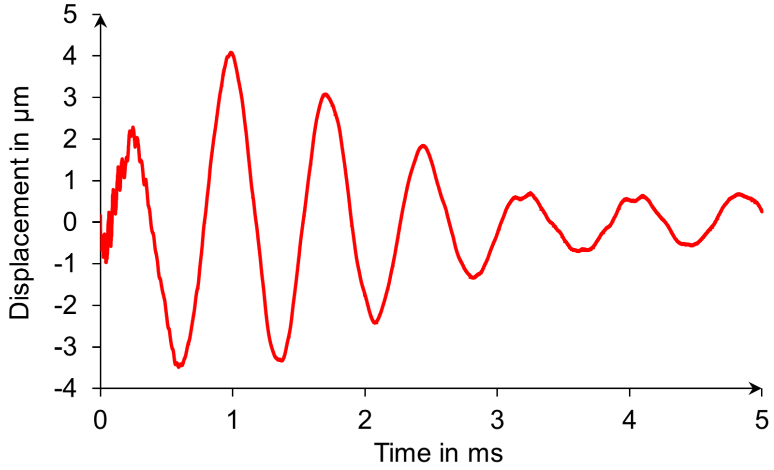

Supplementary Fig. 25: Surface displacement of the meteorite sample measured by LDV. The plot shows the displacement (in µm) as a function of time (in ms) for shot 26 with a beam intensity of 3×10^{11} protons.

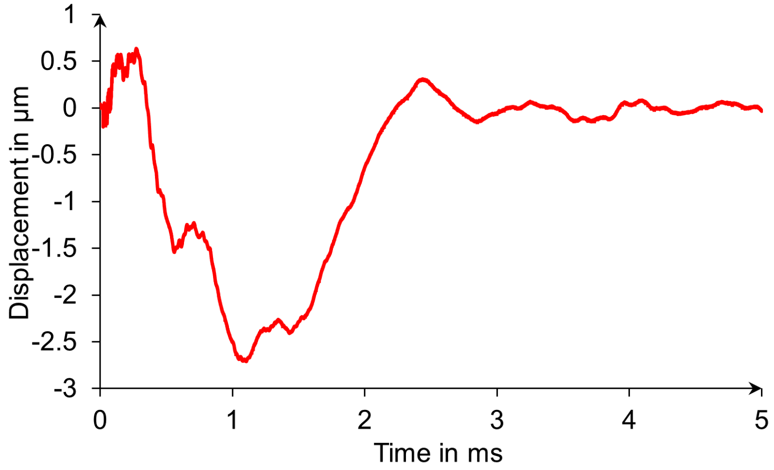

Supplementary Fig. 26: Surface displacement of the meteorite sample measured by LDV. The plot shows the displacement (in µm) as a function of time (in ms) for shot 27 with a beam intensity of 1×10^{11} protons.

Supplementary Figures 27–50 present the Fast Fourier Transform (FFT) spectra obtained for each corresponding beam shot

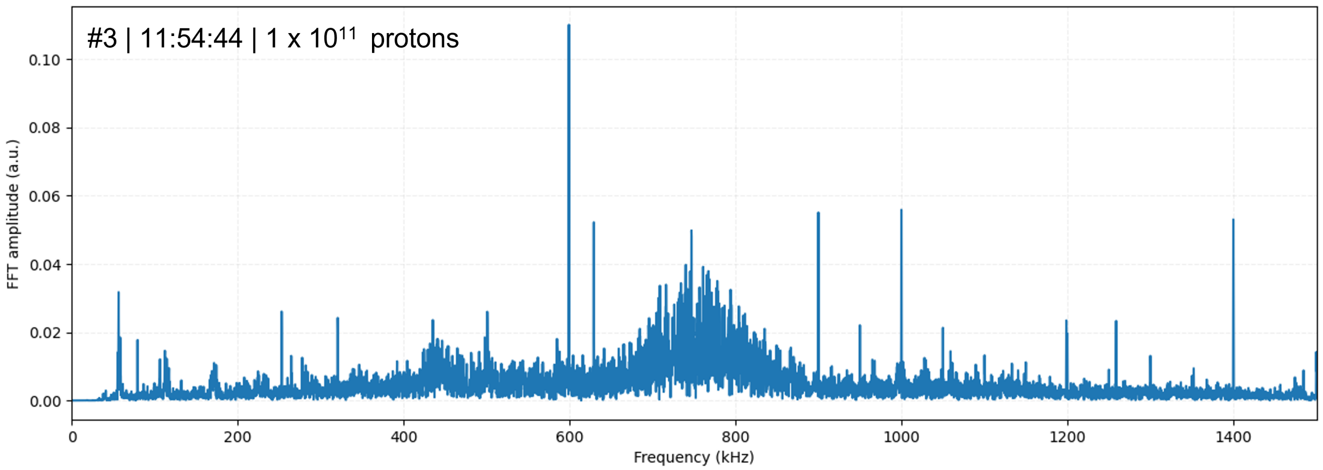

Supplementary Fig. 27: Fast Fourier Transform (FFT) spectrum of the meteorite sample oscillations at beam shot 3 with a beam intensity of 1×10^{11} protons.

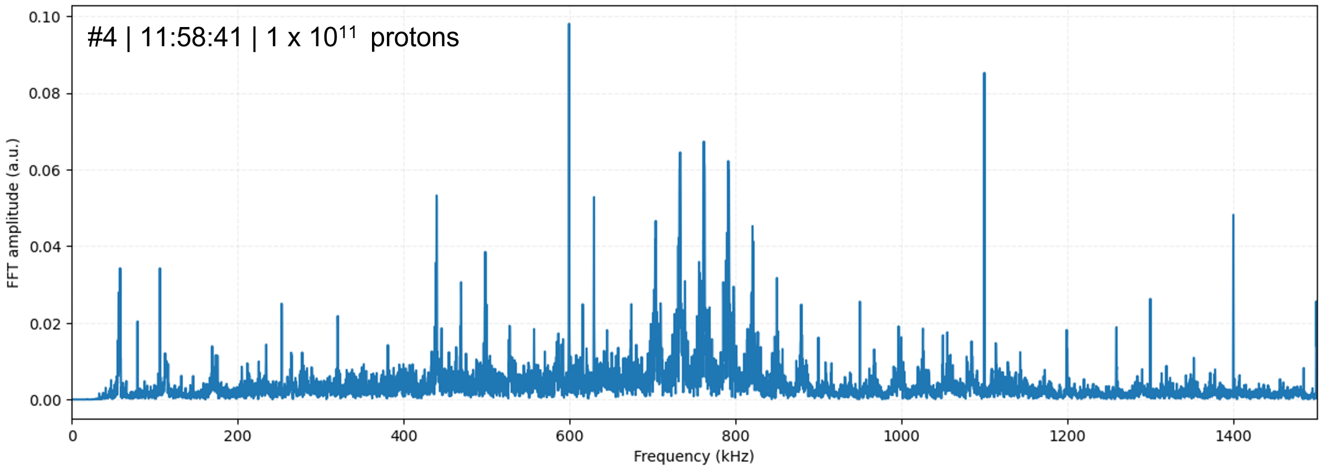

Supplementary Fig. 28: Fast Fourier Transform (FFT) spectrum of the meteorite sample oscillations at beam shot 4 with a beam intensity of 1×10^{11} protons.

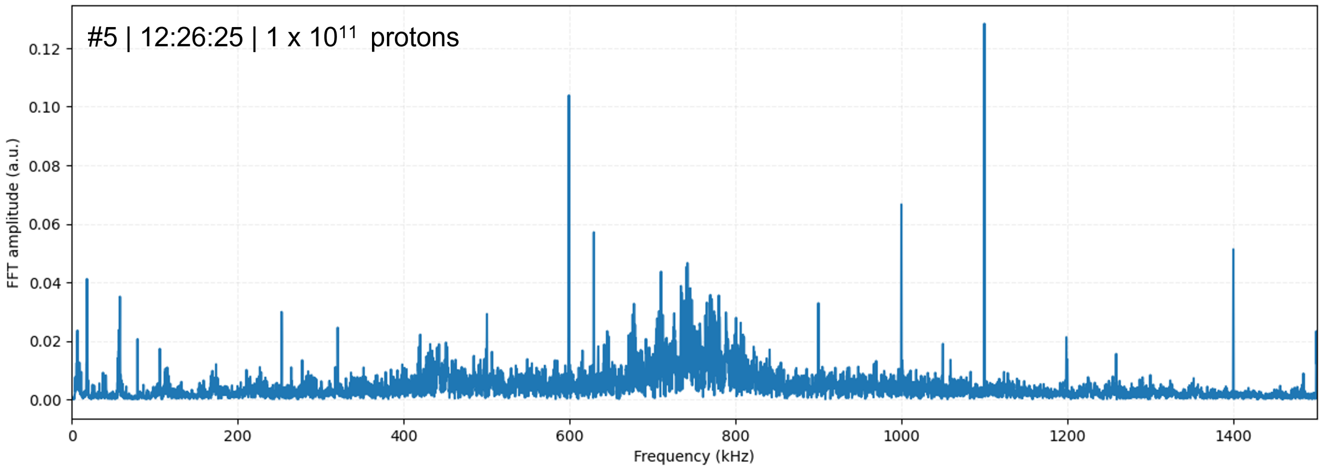

Supplementary Fig. 29: Fast Fourier Transform (FFT) spectrum of the meteorite sample oscillations at beam shot 5 with a beam intensity of 1×10^{11} protons.

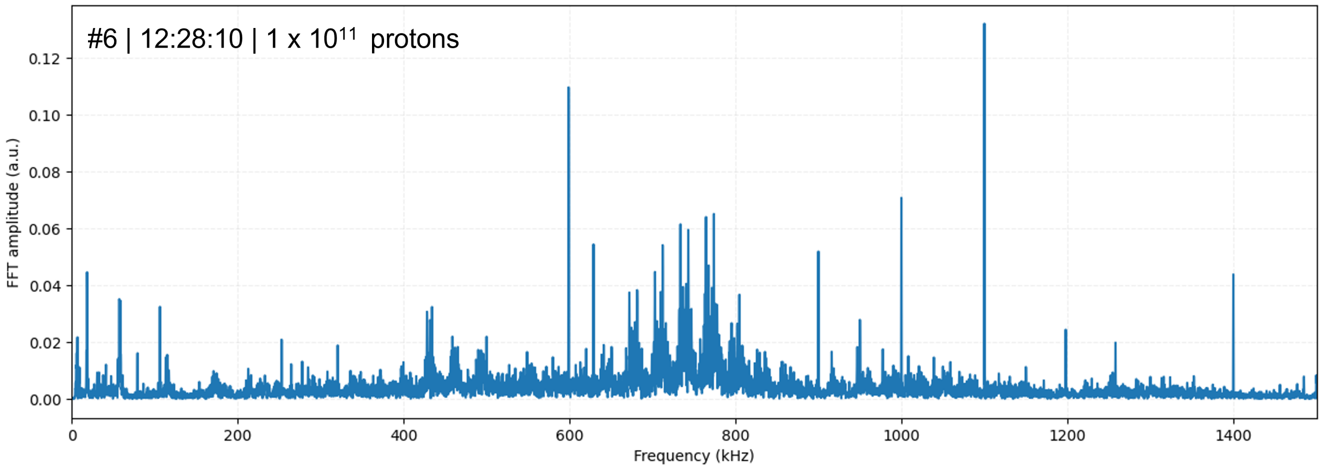

Supplementary Fig. 30: Fast Fourier Transform (FFT) spectrum of the meteorite sample oscillations at beam shot 6 with a beam intensity of 1×10^{11} protons.

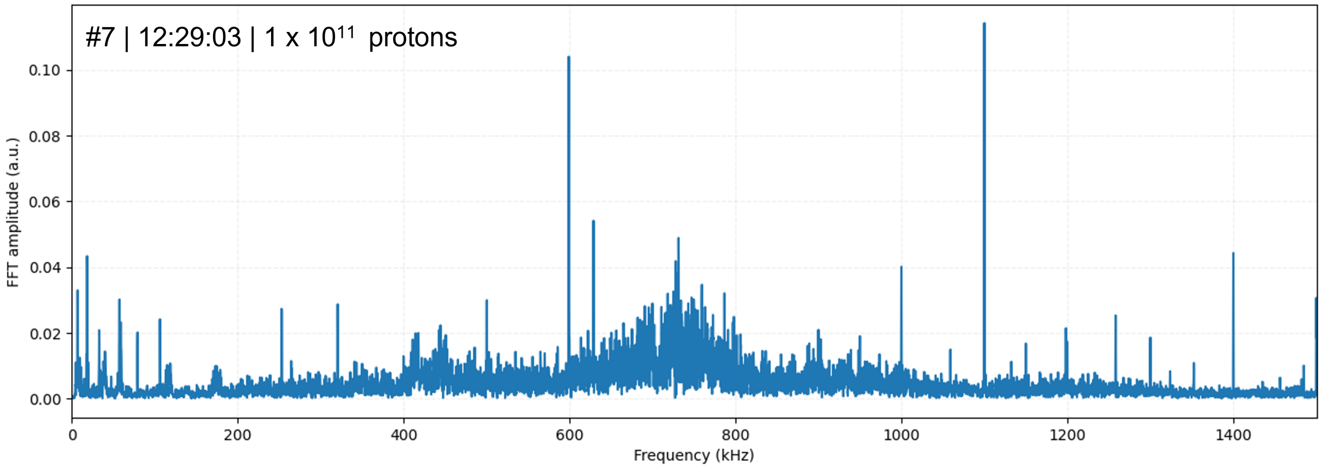

Supplementary Fig. 31: Fast Fourier Transform (FFT) spectrum of the meteorite sample oscillations at beam shot 7 with a beam intensity of 1×10^{11} protons.

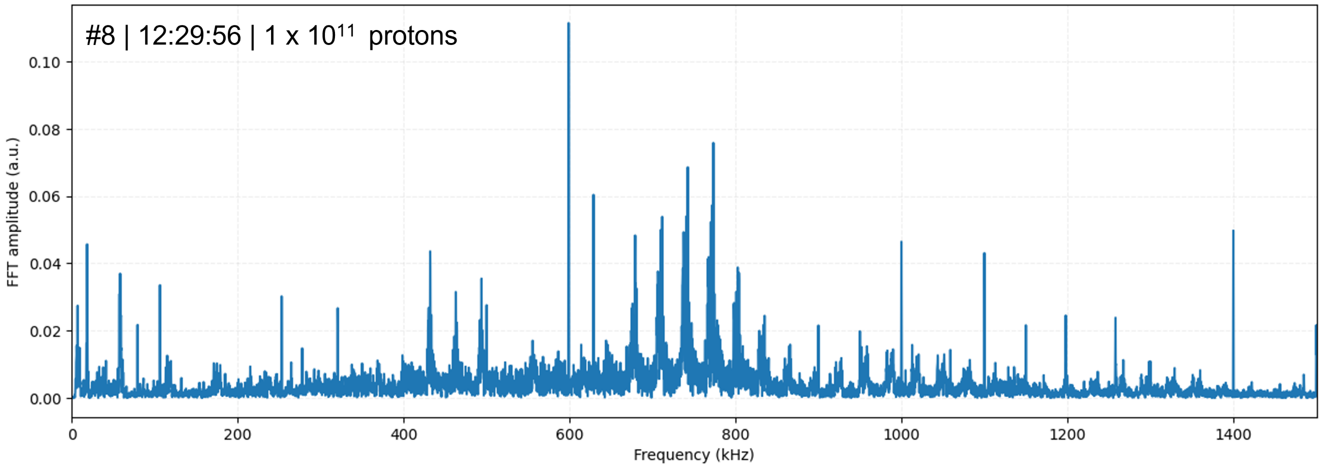

Supplementary Fig. 32: Fast Fourier Transform (FFT) spectrum of the meteorite sample oscillations at beam shot 8 with a beam intensity of 1×10^{11} protons.

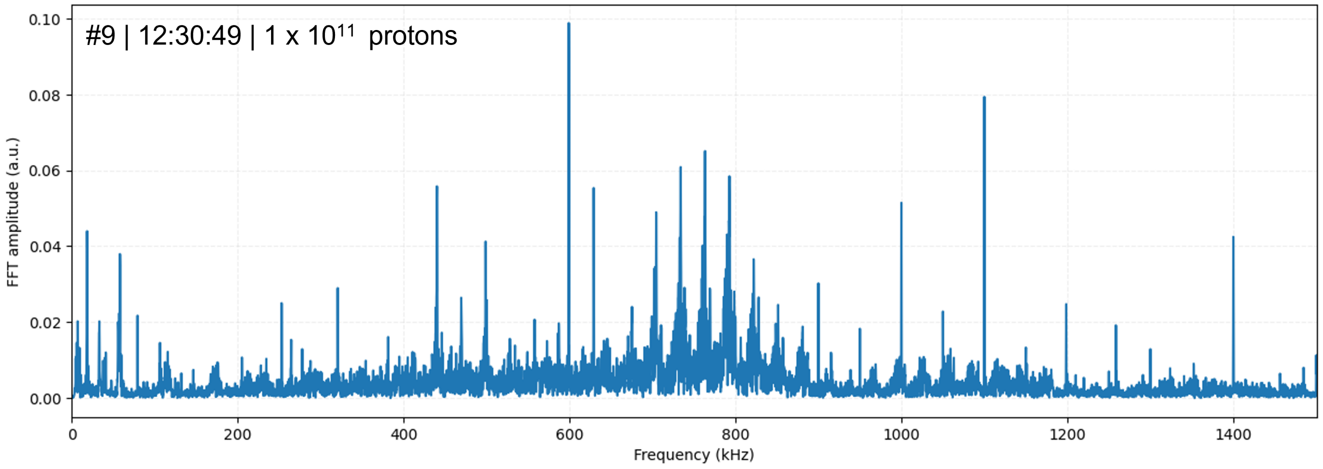

Supplementary Fig. 33: Fast Fourier Transform (FFT) spectrum of the meteorite sample oscillations at beam shot 9 with a beam intensity of 1×10^{11} protons.

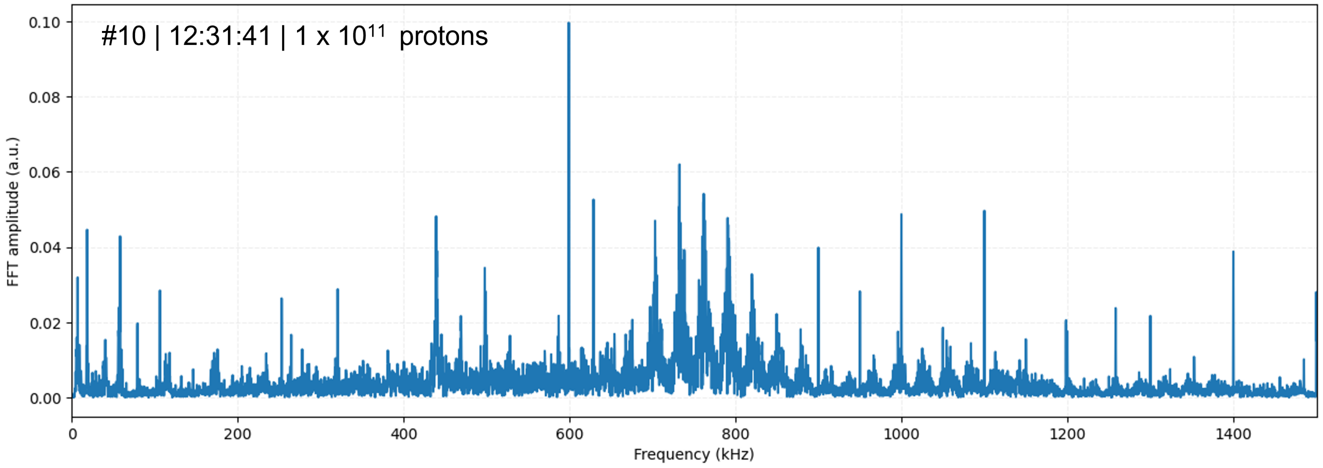

Supplementary Fig. 34: Fast Fourier Transform (FFT) spectrum of the meteorite sample oscillations at beam shot 10 with a beam intensity of 1×10^{11} protons.

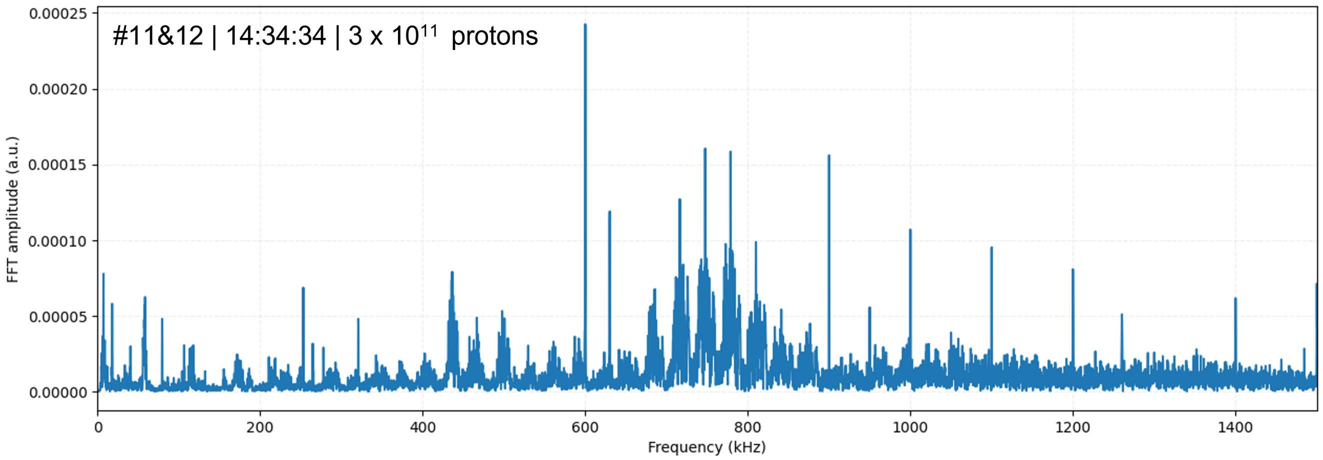

Supplementary Fig. 35: Fast Fourier Transform (FFT) spectrum of the meteorite sample oscillations at beam shot 11&12 with a beam intensity of 3×10^{11} protons.

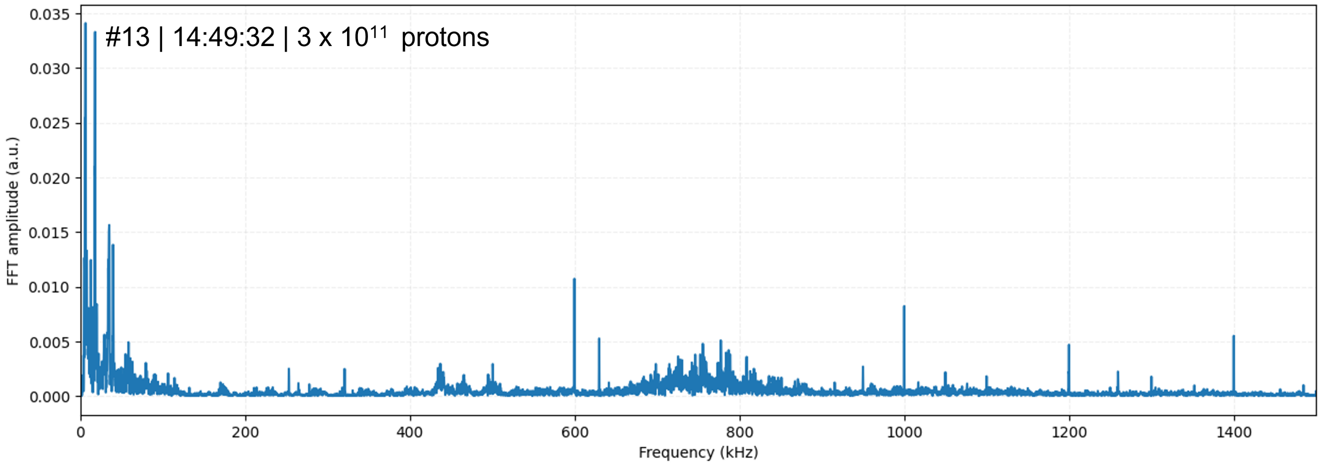

Supplementary Fig. 36: Fast Fourier Transform (FFT) spectrum of the meteorite sample oscillations at beam shot 13 with a beam intensity of 3×10^{11} protons.

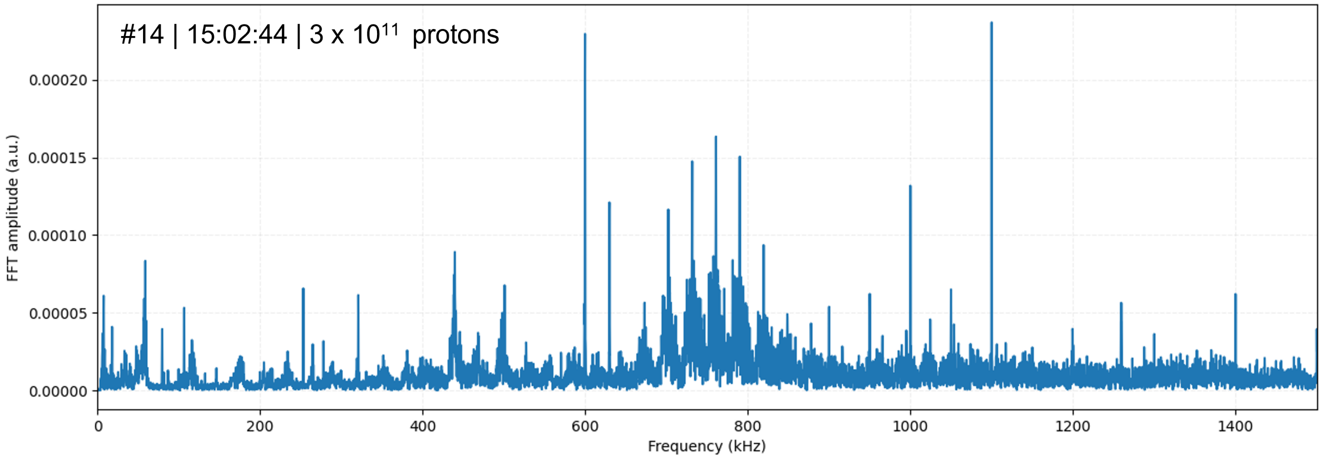

Supplementary Fig. 37: Fast Fourier Transform (FFT) spectrum of the meteorite sample oscillations at beam shot 14 with a beam intensity of 3×10^{11} protons.

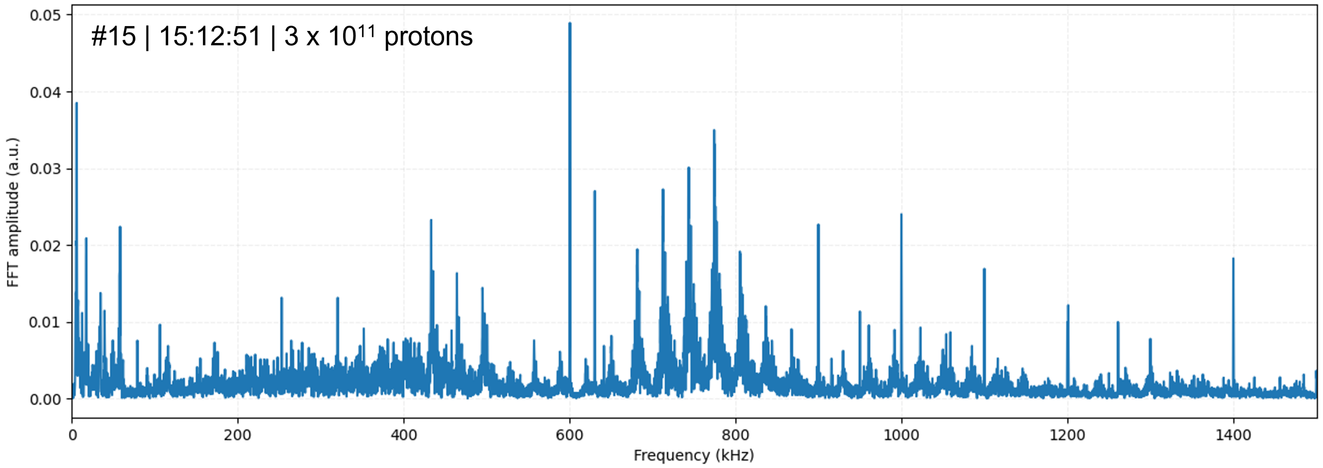

Supplementary Fig. 38: Fast Fourier Transform (FFT) spectrum of the meteorite sample oscillations at beam shot 15 with a beam intensity of 3×10^{11} protons.

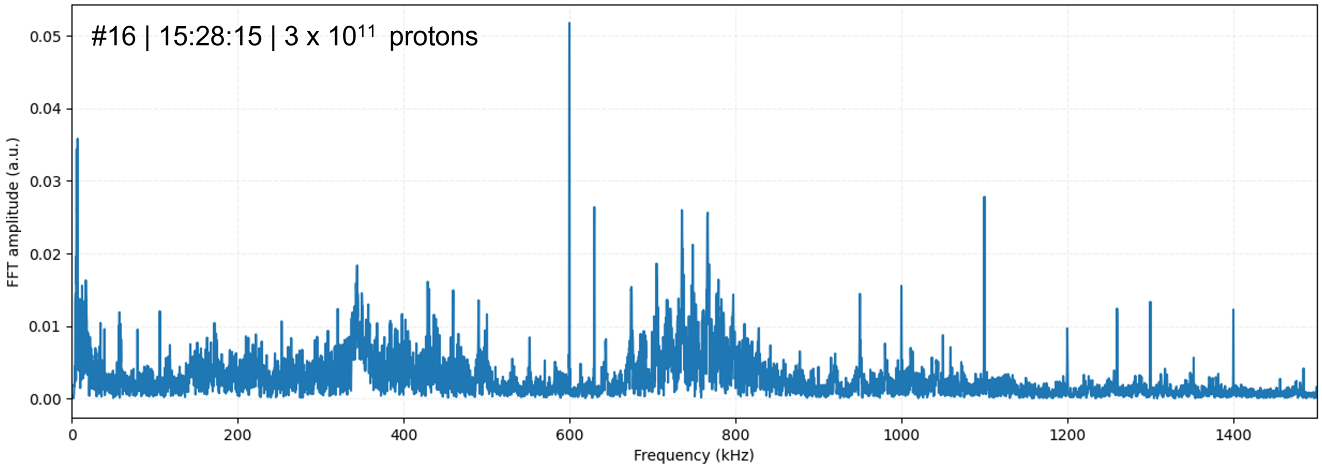

Supplementary Fig. 39: Fast Fourier Transform (FFT) spectrum of the meteorite sample oscillations at beam shot 16 with a beam intensity of 3×10^{11} protons.

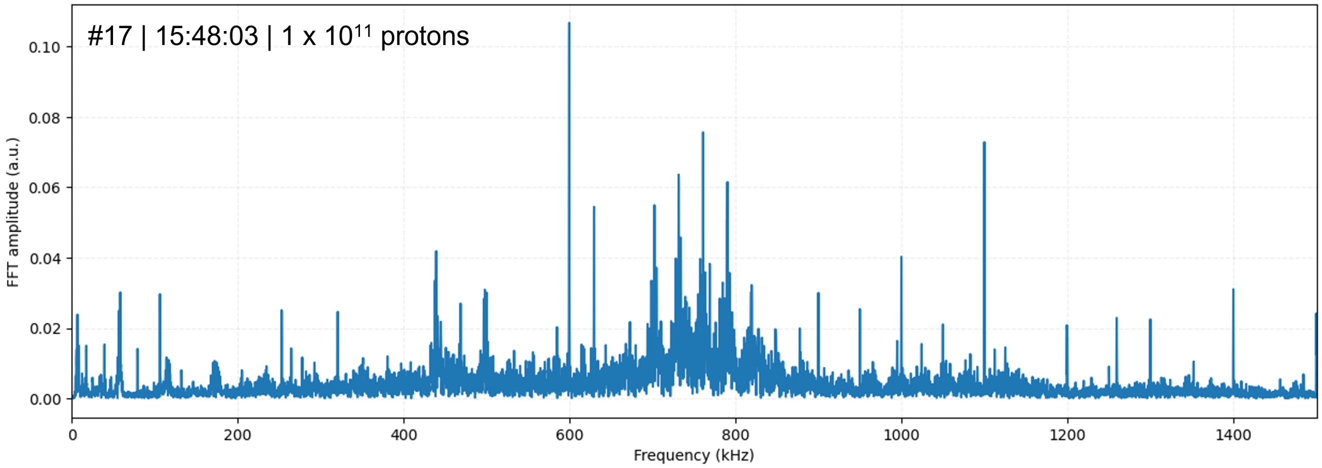

Supplementary Fig. 40: Fast Fourier Transform (FFT) spectrum of the meteorite sample oscillations at beam shot 17 with a beam intensity of 1×10^{11} protons.

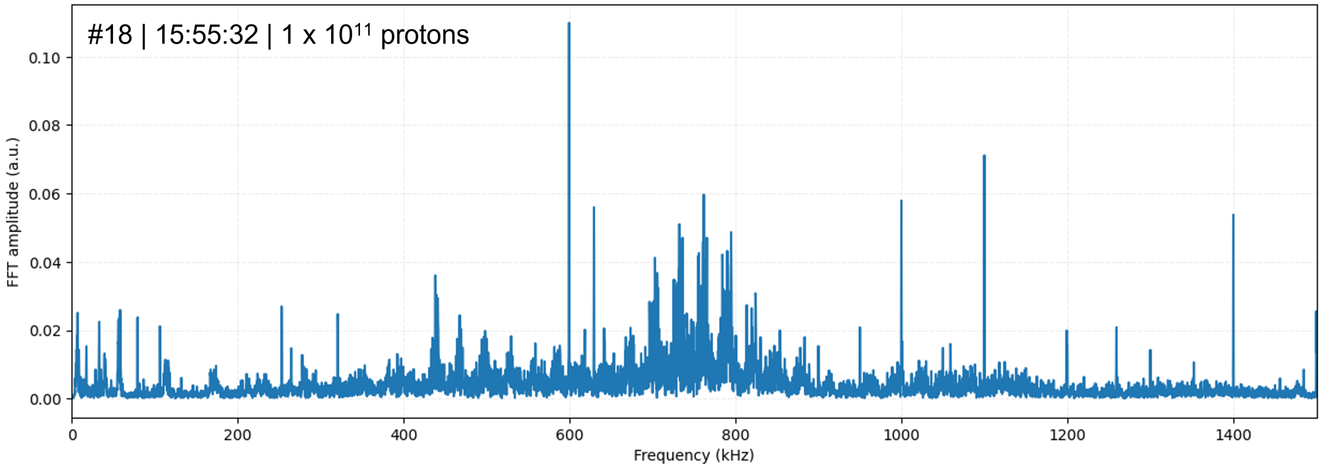

Supplementary Fig. 41: Fast Fourier Transform (FFT) spectrum of the meteorite sample oscillations at beam shot 18 with a beam intensity of 1×10^{11} protons.

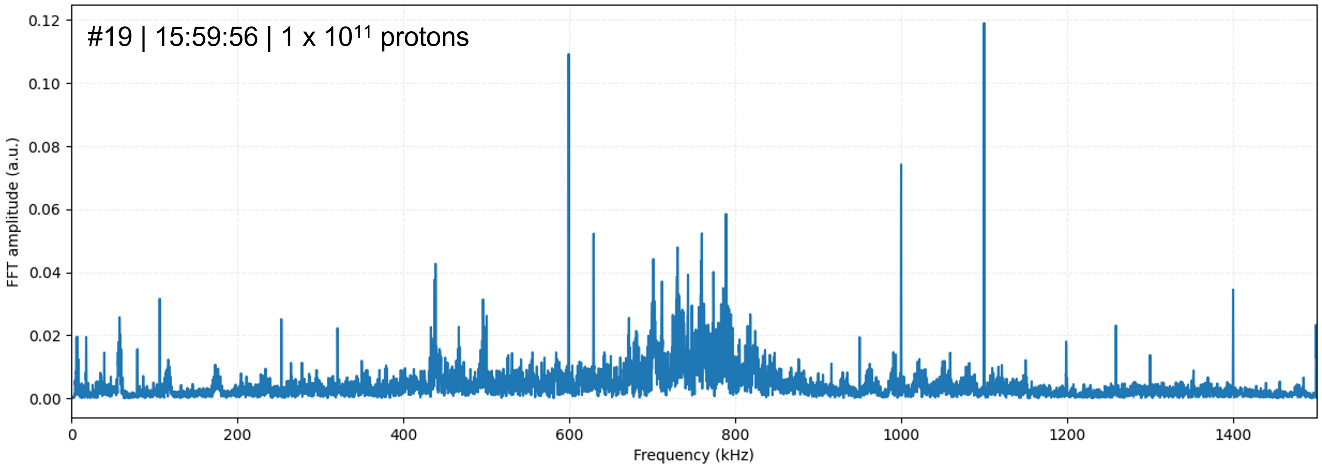

Supplementary Fig. 42: Fast Fourier Transform (FFT) spectrum of the meteorite sample oscillations at beam shot 19 with a beam intensity of 1×10^{11} protons.

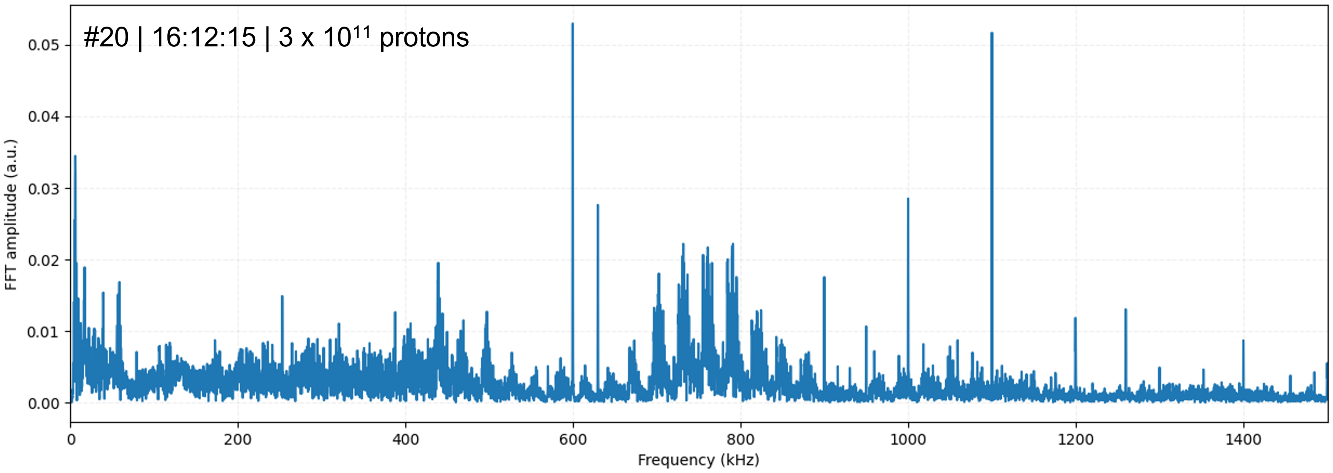

Supplementary Fig. 43: Fast Fourier Transform (FFT) spectrum of the meteorite sample oscillations at beam shot 20 with a beam intensity of 3×10^{11} protons.

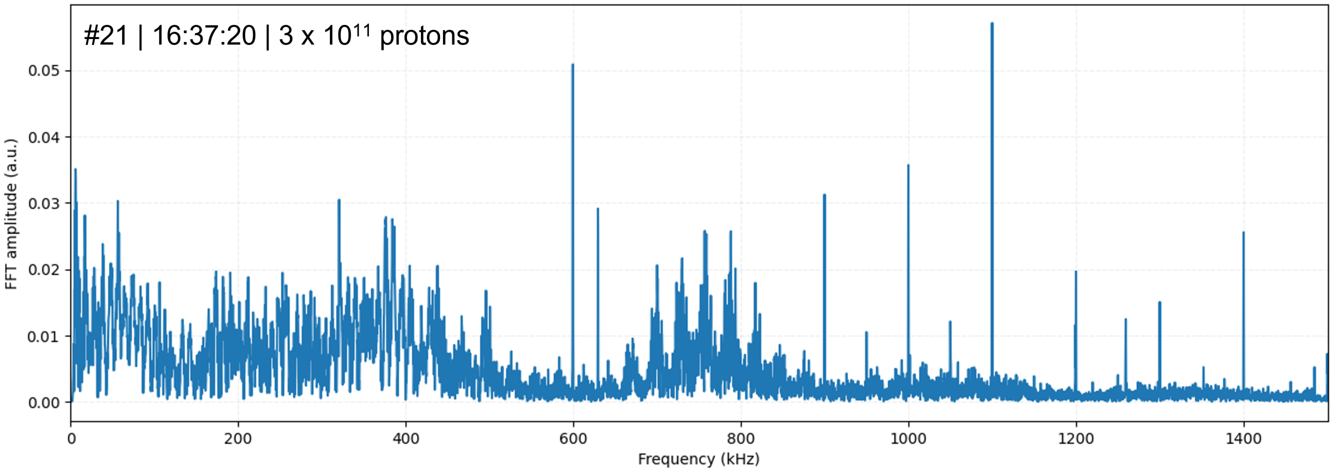

Supplementary Fig. 44: Fast Fourier Transform (FFT) spectrum of the meteorite sample oscillations at beam shot 21 with a beam intensity of 3×10^{11} protons.

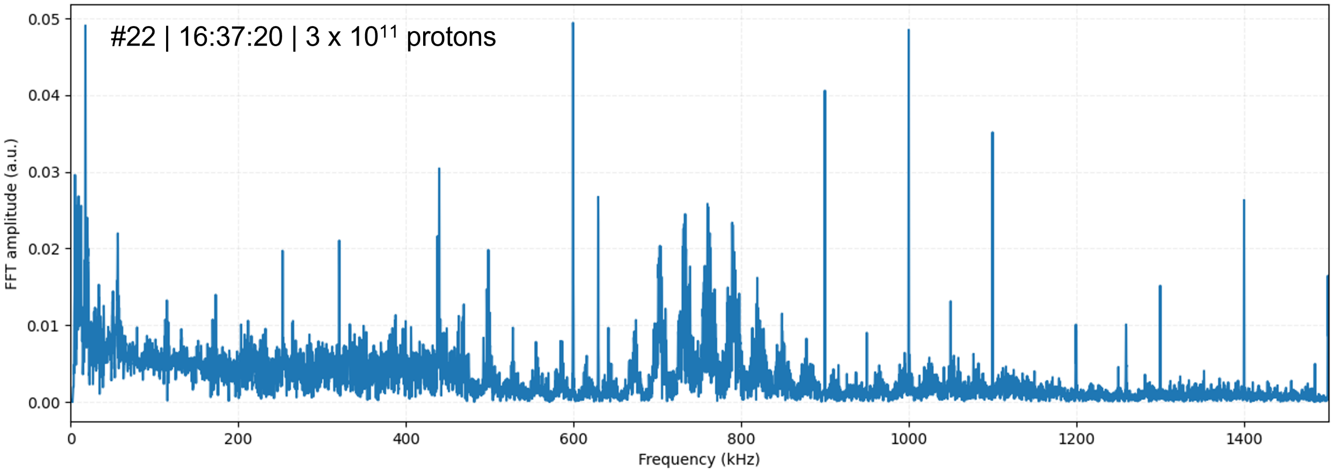

Supplementary Fig. 45: Fast Fourier Transform (FFT) spectrum of the meteorite sample oscillations at beam shot 22 with a beam intensity of 3×10^{11} protons.

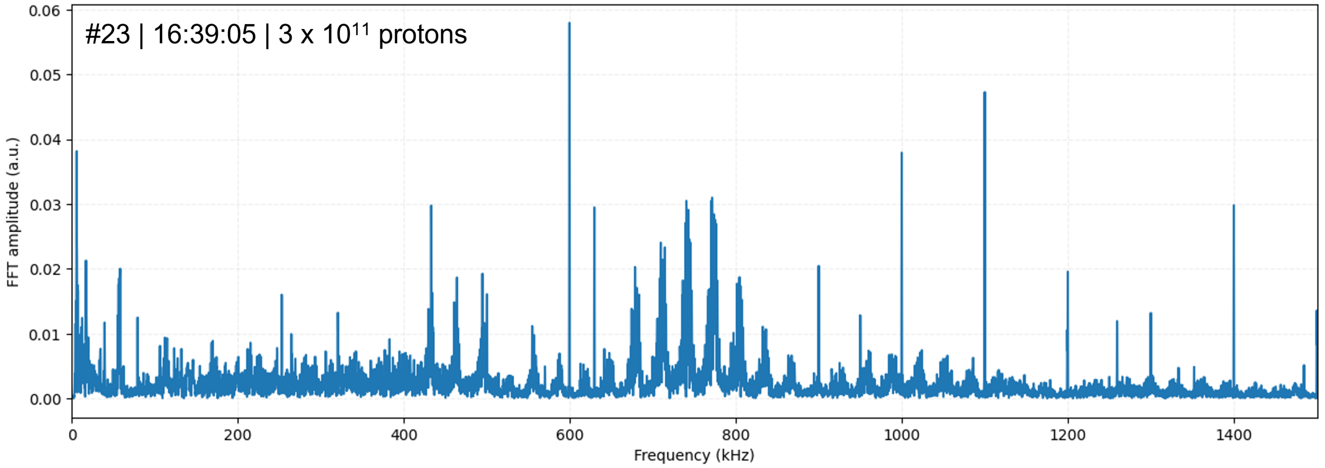

Supplementary Fig. 46: Fast Fourier Transform (FFT) spectrum of the meteorite sample oscillations at beam shot 23 with a beam intensity of 3×10^{11} protons.

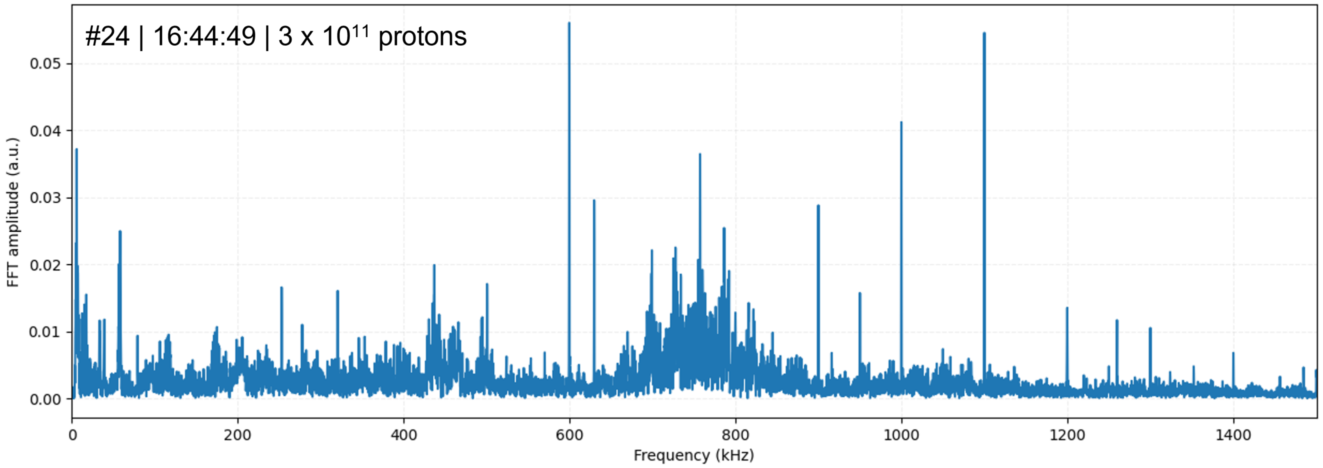

Supplementary Fig. 47: Fast Fourier Transform (FFT) spectrum of the meteorite sample oscillations at beam shot 24 with a beam intensity of 3×10^{11} protons.

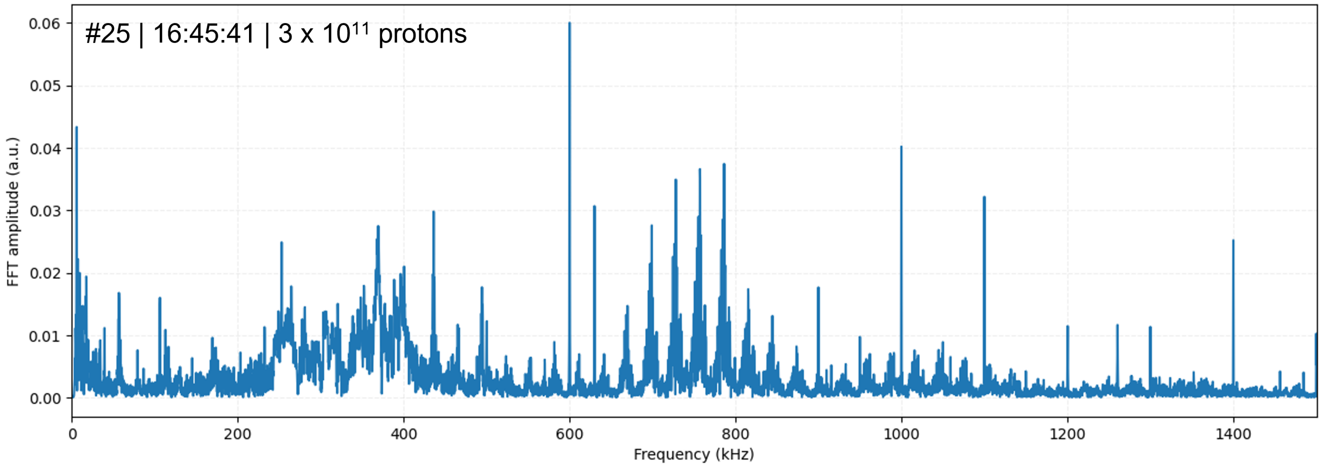

Supplementary Fig. 48: Fast Fourier Transform (FFT) spectrum of the meteorite sample oscillations at beam shot 25 with a beam intensity of 3×10^{11} protons.

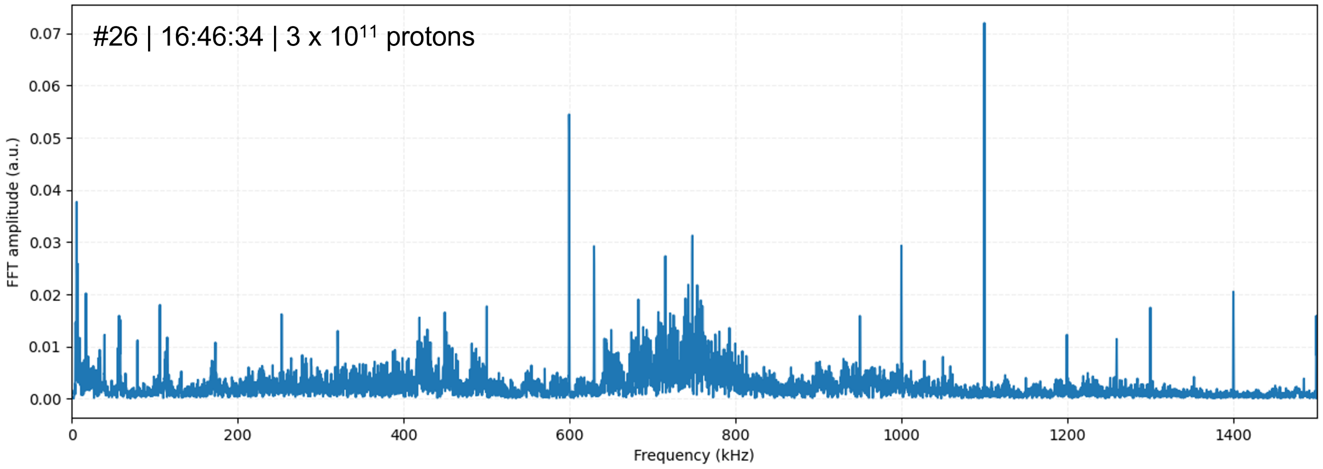

Supplementary Fig. 49: Fast Fourier Transform (FFT) spectrum of the meteorite sample oscillations at beam shot 26 with a beam intensity of 3×10^{11} protons.

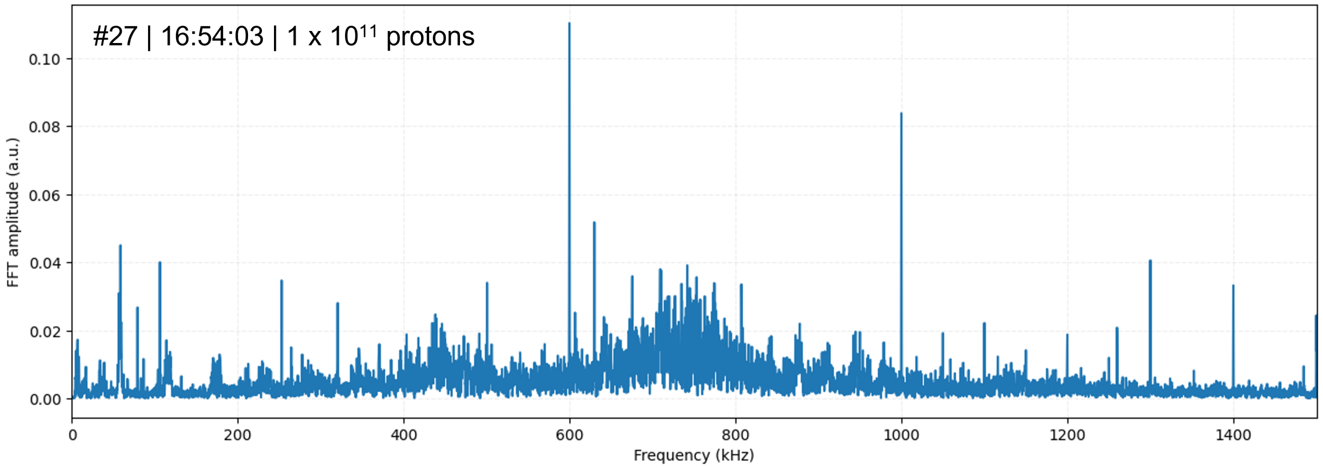

Supplementary Fig. 50: Fast Fourier Transform (FFT) spectrum of the meteorite sample oscillations at beam shot 27 with a beam intensity of 1×10^{11} protons.

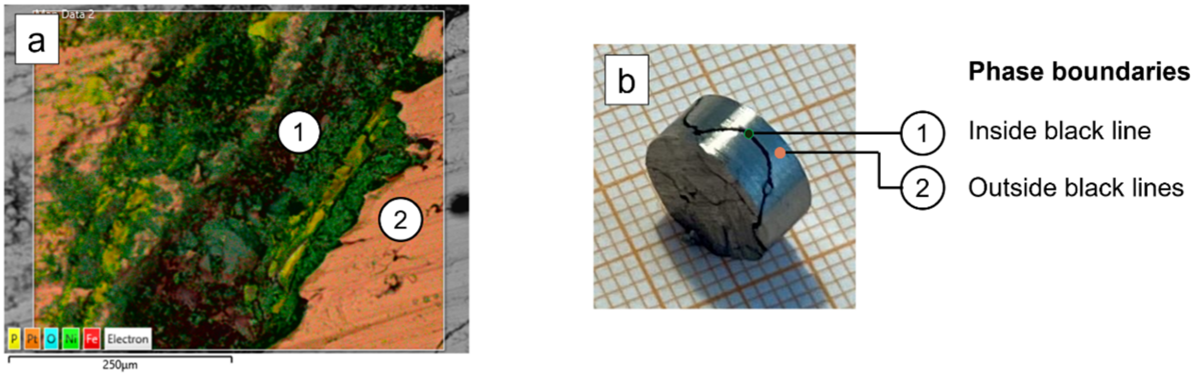

Supplementary Fig. 51: Phase boundary of Campo del Cielo meteorite a) SEM-image of phase boundary, b) macroscopic picture of exemplary phase boundary of Campo del Cielo iron meteorite.
