# Supplementary material for: Dynamical development of strength and stability of asteroid material under 440 GeV proton beam irradiation
Source: Nat Commun. 2025 Nov 28;16:11710. doi: 10.1038/s41467-025-66912-4 (PMC12753701; doi:10.1038/s41467-025-66912-4)
Supplement: Supplementary file 2 — Description of Additional Supplementary Files [file 41467_2025_66912_MOESM2_ESM.pdf]

Description of Additional Supplementary Files

File Name	Description
`Supplementary Data 1.dat`	Contains the source data for **Figure 1**, specifically the energy deposition data of the iron meteorite sample.
`Supplementary Data 2.xls`	Contains the source data for Figures 2, 3, and 5.<br><br>Explanation of worksheet tabs:<br>• **Fig2_Source data**: Pulse list with beam parameters.<br>• **Fig3a_Source data**: Displacement data for oscillating beam shots (#3–10).<br>• **Fig3b_Source data**: Displacement data for non-oscillating beam shots (#13–22).<br>• **Fig3c_Source data**: Displacement data for oscillating beam shots (#11–12, 23, 25, 26).<br>• **Fig5_Source data**: Input data for Figure 5.
